# Differentially-expressed genes in rice infected by *Xanthomonas oryzae* pv. *oryzae* relative to a flagellin-deficient mutant reveal potential functions of flagellin in host–pathogen interactions

**DOI:** 10.1186/s12284-014-0020-7

**Published:** 2014-09-03

**Authors:** Chao Yu, Huamin Chen, Fang Tian, Jan E Leach, Chenyang He

**Affiliations:** 1 State Key Laboratory for Biology of Plant Diseases and Insect Pests, Institute of Plant Protection, Chinese Academy of Agricultural Sciences, Beijing 100193, China; 2 Department of Bioagricultural Sciences and Pest Management, Colorado State University, Ft. Collins 80523-1177, CO, USA

**Keywords:** Rice, Differentially-expressed genes (DEGs), Flagellin, Immune response, Xanthomonas oryzae pv. oryzae

## Abstract

**Background:**

Plants have evolved a sensitive defense response system that detects and recognizes various pathogen-associated molecular patterns (PAMPs) (e.g. flagellin) and induces immune responses to protect against invasion. Transcriptional responses in rice to PAMPs produced by *Xanthomonas oryzae* pv. *oryzae* (Xoo), the bacterial blight pathogen, have not yet been defined.

**Results:**

We characterized transcriptomic responses in rice inoculated with the wildtype (WT) Xoo and flagellin-deficient mutant *∆fliC* through RNA-seq analysis. Digital gene expression (DGE) analysis based on Solexa/Illumina sequencing was used to investigate transcriptomic responses in 30 day-old seedlings of rice (*Oryza sativa* L. cv. Nipponbare). 1,680 genes were differentially-expressed (DEGs) in rice inoculated with WT relative to ∆*fliC*; among which 1,159 genes were up-regulated and 521 were down-regulated. Expression patterns of 12 randomly-selected DEGs assayed by quantitative real time PCR (qRT-PCR) were similar to those detected by DGE analyses, confirming reliability of the DGE data. Functional annotations revealed the up-regulated DEGs are involved in the cell wall, lipid and secondary metabolism, defense response and hormone signaling, whereas the down-regulated ones are associated with photosynthesis. Moreover, 57 and 21 specifically expressed genes were found after WT and ∆*fliC* treatments, respectively.

**Conclusions:**

DEGs were identified in rice inoculated with WT Xoo relative to ∆*fliC*. These genes were predicted to function in multiple biological processes, including the defense response and photosynthesis in rice. This study provided additional insights into molecular basis of rice response to bacterial infection and revealed potential functions of bacterial flagellin in the rice-Xoo interactions.

## Background

Plants, unlike mammals, do not have an adaptive immunity mechanism, and largely rely on the innate immune system to resist pathogen infection (Jones and Dangl [2006]). Pattern-triggered immunity (PTI) and effector-triggered immunity (ETI) are two layers of innate immune system in plant (Chisholm et al. [2006]; Tsuda and Katagiri [2010]). Pathogen-associated molecular patterns (PAMPs) are conserved and indispensable molecules, which include bacterial flagellin, elongation factor-Tu (EF-Tu), fungal glucans, chitin, etc. (Zhang and Zhou [2010]). PTI is activated through recognition of PAMPs by pattern recognition receptors (PRRs) located on the plant cell surface; the resulting basal defense response prevents potential pathogens from colonizing on plant tissues (Chakravarthy et al. [2009]; Chujo et al. [2013]). PTI is characterized by induction of reactive oxygen species (ROS), ion fluxes, callose deposition in the cell wall and other innate immune responses (Tsuda et al. [2008]; Boller and Felix [2009]). During the evolution of plant-pathogen interactions, some pathogens acquired the ability to suppress PTI by delivering effectors to plant cells, thus causing disease (Li et al. [2005]; Chisholm et al. [2006]). Then plants developed resistance (R) proteins to recognize effectors, leading to activation of effector-triggered immunity (ETI) (Jones and Dangl [2006]). Pathogenesis related proteins (PRs) and WRKY transcription factors (WRKY TFs) are two kinds of important proteins involved in defense to pathogens in both PTI and ETI (Chujo et al. [2013]).

Flagellin, the main component of a bacterial flagellum, was first identified as an elicitor that induces defense responses in tomato and *Arabidopsis thaliana* (Felix et al. [1999]). Flg22, a 22 amino acid, highly conserved domain within the N terminus of flagellin, caused these defense responses and a strong inhibition of growth in *A. thaliana* seedlings (Gomez-Gomez et al. [1999]). FLAGELLIN SENSITIVE2 (FLS2), the receptor that recognizes flagellin in *A. thaliana*, shares structural and functional homologies with known plant resistance proteins (Gomez-Gomez and Boller [2000]). Flagellin and flg22 are recognized and bound to the extracelluar domain of FLS2. This induces FLS2 heteromerization with BRASSINOSTEROID INSENSITIVE 1-associate kinase 1 (BAK1) and activates signaling cascades that finally culminate in the induction of defense responses (Heese et al. [2007]; Ali and Reddy [2008]; Sun et al. [2013]). Therefore, FLS2 determined the specificity of flagellin perception in *A. thaliana* (Chinchilla et al. [2006]). OsFLS2, the ortholog of FLS2 in rice, also mediates immune responses induced by flagellin (Takai et al. [2008]).

Different responses of rice cells to flagellins from compatible or incompatible bacterial strains have been observed. For example, flagellin purified from an incompatible strain of *Acidovorax avenae* (N1141), induced the rapid generation of H_2_O_2_ accompanying hypersensitive cell death and the expression of *PAL*, *Cht-1*,and *PBZ1* in cultured rice cells, whereas the flagellin from the compatible strain K1 did not (Che et al. [2000]; Tanaka et al. [2003]). Introduction of N1141 flagellin gene into rice also trigged immune responses (Takakura et al. [2008]). However, flg22 did not induce defense responses in rice, suggesting that the recognition mechanism for flagellin might be different between rice and dicotyledonous plants, such as Arabidopsis and tomato (Felix et al. [1999]; Takai et al. [2007]). Interestingly, when expressed and purified from *Escherichia coli*, both types of flagellins induced H_2_O_2_ generation in rice. In addition, a deglycosylated flagellin from the compatible strain induced the same immune responses as the flagellin of incompatible strain did (Hirai et al. [2011]). These studies suggested that post-translational modifications of flagellins might be associated with the specific induction of immune responses in rice. However, the molecular mechanism of different immune responses of induced by different flagellins remains largely unknown.

*Xanthomonas oryzae* pv. *oryzae* (Xoo), causes bacterial blight disease and can result in serious yield loss in rice production. The Xoo-rice interaction has been studied as a model system to understand the molecular mechanisms of disease resistance responses in monocotyledonous plants (Song et al. [1995]; Ronald [1997]; Martin et al. [2003]). Microarray studies revealed that several signaling components, membrane bound receptor kinases and disease-resistant proteins were significantly induced after Xoo inoculation (Narsai et al. [2013]). However, the function of flagellin in Xoo-rice interaction has never been studied. In a previous study, we generated a mutant of Xoo that was deleted in the flagellin gene *fliC* (∆*fliC*); the ∆*fliC* mutant was not motile, and caused more disease (increased lesion lengths) in rice leaves relative to the wildtype (WT) strain (Tian et al. [2014]), suggesting that differential rice responses to the WT and ∆*fliC* inoculations exist.

In this study, digital gene expression (DGE) based on Solexa/Illumina sequencing, was applied to identify differentially-expressed genes (DEGs) in rice inoculated with WT Xoo relative to the ∆*fliC* mutant. We identified 1,680 DEGs involved in cell wall and lipid synthesis, secondary metabolism, photosynthesis, defense response, and hormone signaling pathways.

## Results

### DGE sequencing in rice leaves inoculated with WT Xoo or ∆*fliC*

For DGE sequencing, RNAs from rice leaves inoculated with the WT or ∆*fliC* mutant were extracted to prepare two cDNA libraries for RNA-seq analysis. After filtering to remove reads containing the adapter and poly-N or low quality reads, 36,187,662 and 38,239,937 clean reads remained in the WT and ∆*fliC* libraries, respectively. The sequencing depth was sufficient for the transcriptome coverage in rice (Table 1).

**Table 1:** Summary of sequencing data

**Sample**	**Raw tag**	**Clean tag**		**GC (%)**	**Tags mapped to gene**	
		**TN**	**TP(%)**		**TN**	**TP(%)**
WT	40,067,282	38,239,937	95.44	54.93	32,279,312	84.41
∆*fliC*	37,643,149	36,187,662	96.13	52.53	30,423,983	84.07

TN: total number; TP: total percentage.

A total of 30,423,983 (84.07%) and 32,279,312 (84.41%) clean reads in the two libraries were mapped to the reference genome of rice using bowtie software and allowing a 2-bp mismatch. The GC contents were 52.53% and 54.93%, respectively (Table 1). Over 80% clean tags per library were mapped to the reference database, showing the DGE data was reliable and sufficient for subsequent bioinformatics analysis of gene expression.

The raw sequencing data obtained in this work have been deposited in NCBI’s Sequence Read Archive (SRA) and are accessible through SRA Series accession number PRJNA238154, and the accession numbers of WT and ∆*fliC* libraries are SRR1168425 and SRR1168426, respectively (http://www.ncbi.nlm.nih.gov/bioproject/?term=PRJNA238154).

### Identification of DEGs in rice inoculated with WT Xoo relative to ∆*fliC*

**Figure 1:**
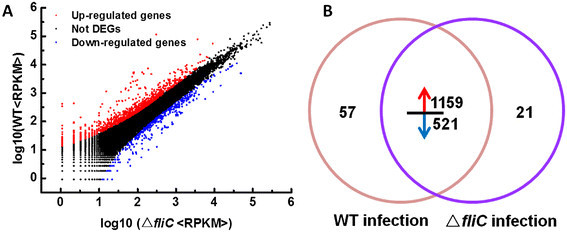
**Differentially-expressed genes (DGEs) between the WT and Δ** ***fliC*** **treatments in rice. A**, Comparison of gene expression levels between WT and ∆*fliC* treatments. Red and blue dots represent transcripts more prevalent in WT and ∆*fliC* libraries, respectively. Black dots indicate transcripts that did not change significantly. The parameters “P ≤ 0.05” and “|log_2_ Ratio| ≥ 1” were used as the threshold to judge the significance of gene expression difference. **B**, Changes in gene expression between the WT and ∆*fliC* libraries. Using the ∆*fliC* library as control, red and blue arrows represent genes were up-regulated and down-regulated in WT library, respectively. Data represent the number of DEGs.

To compare differential gene transcription in WT- and ∆*fliC*-treated rice, two transcriptome profiles were analyzed. As described in materials and methods, the number of clean reads in each library was normalized to the number of transcripts per kilobase of exon model per million mapped reads (FPKM) to obtain the normalized gene expression level, then the relative expression levels were calculated by taking ∆*fliC* treatment as the control. Among the 1,680 DEGs identified, 1,159 were up-regulated and 521 were down-regulated in the WT relative to ∆*fliC* treatments (Figure 1). In addition, 57 and 21 DEGs were specifically expressed in WT and ∆*fliC* libraries, respectively (Figure 1B).

**Figure 2:**
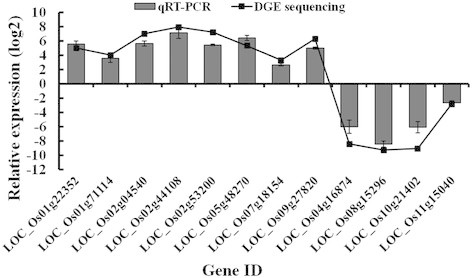
**Confirmation of expression of 12 randomly-selected DEGs by qRT-PCR assays.** Lines indicate expression from DEG analysis and columns indicate relative expression as measured by qRT-PCR. Bars indicate standard error.

To validate the reliability of DGE sequencing, the expression of 12 randomly-selected DEGs were revealed by quantitative real time PCR (qRT-PCR) assays in another independent inoculation experiment. The results showed all of gene expression patterns were consistent to those from the DGE analysis, indicating the DGE data was reliable (Figure 2).

### Functional classifications of DEGs

**Figure 3:**
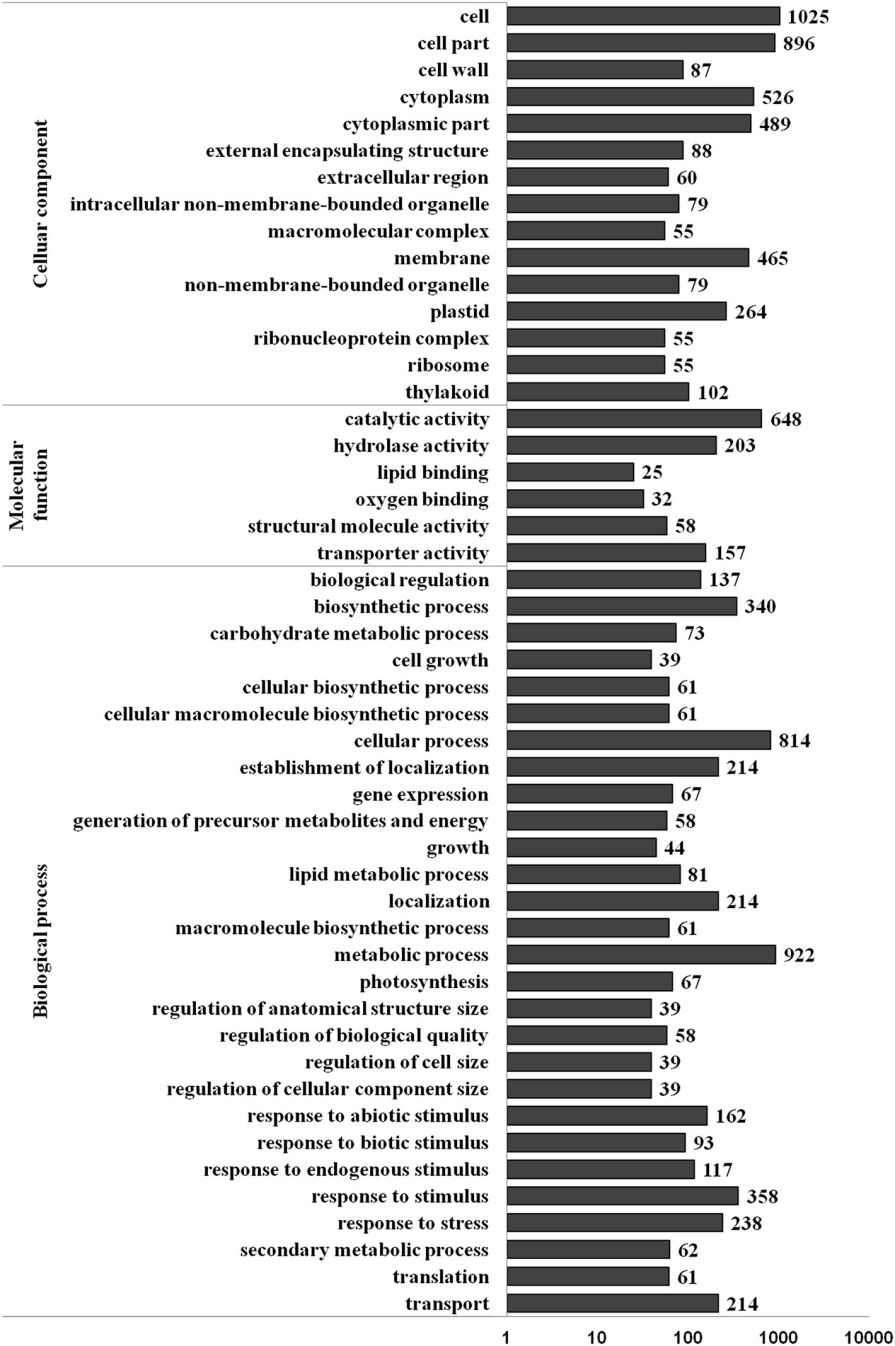
**Histogram showing gene ontology functional analysis of DEGs.** The y-axis and X-axis indicated the names of clusters and the number of DEGs in clusters, respectively.

A total of 1,758 DEGs between WT and ∆*fliC* treatments were categorized into 49 functional groups using GO classifications (Figure 3). In the biological process category, metabolic process was the most dominant group, followed by cellular process, response to stimulus and biosynthetic process. Additionally, there were many DEGs involved in carbohydrate metabolism, photosynthesis and secondary metabolism. Among the cellular components, cell was the largest group, followed by cell part and cytoplasm. Catalytic activity was the largest group in molecular function, followed by hydrolase activity, and transporter activity. These results indicate that the bacterial flagellin influences expression of genes involved in multiple physiological and biochemical activities in rice.

### Metabolism pathway analysis of DEGs

**Figure 4:**
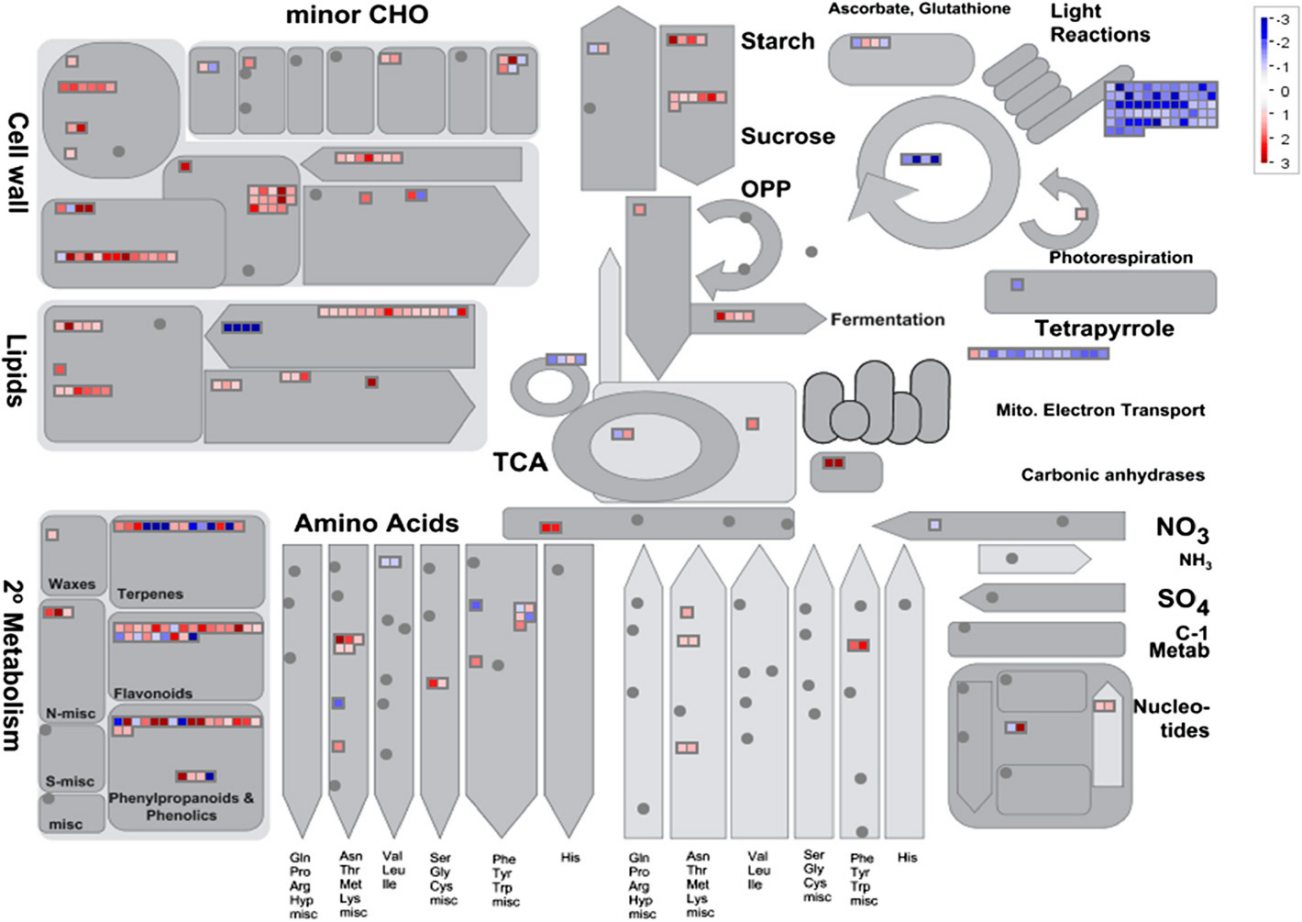
**Metabolism overview between WT and Δ** ***fliC*** **treatments by MapMan.** 1,680 DGEs (fold changes ≥ 2, p-value ≤ 0.05) were imported into MapMan. DGEs up- and down-regulated are represented with red and blue squares, respectively. The grey circles means no differentially-expressed genes involved in this process. Values are log_2_-transformed fold changes.

To further reveal the potential functions of DEGs, an overview of the metabolic processes in rice transcriptionally affected by WT and ∆*fliC* infection was obtained by using MapMan (Thimm et al. [2004]). Cell wall, lipids metabolism, secondary metabolism, amino acid synthesis and degradation, photosynthesis, and other metabolic processes were detected (Figure 4). The DEGs associated with photosynthesis showed significant expression differences between WT- and Δ*fliC*-treated rice. Among them, genes involved in light reactions, Calvin cycle and ATP synthesis were down-regulated, while those involved in starch and sucrose degradation were up-regulated (Additional file 1: Table S5). These results suggest that bacterial flagellin suppresses photosynthetic assimilation in rice.

As the first barrier to prevent pathogen infection in plant, the cell wall is important in plant-pathogen interactions. Most of DEGs associated with cell wall structure were up-regulated in the WT relative to Δ*fliC*-treated rice, including cell wall proteins, cellulose and hemicellulose synthesis and other genes involved in cell wall modification. However, DEGs encoding pectin esterase and pectate lyases were down-regulated (Additional file 2: Table S2).

Lipid metabolism has an important role in defense response to pathogen infection by providing the energy. Five phospholipid synthesis-related DEGs and one glycolipid synthesis-associated DEG were up-regulated in WT relative to Δ*fliC*-treated rice. Most of DEGs involved in fatty acid synthesis and degradation were induced, while four DEGs involved in fatty acid desaturation were significantly repressed (Figure 4). These results indicated that lipid metabolism in rice is accelerated by flagellin.

Secondary metabolism is closely linked with defense responses to biotic stresses in plant. In this study, DEGs involved in terpene, flavonoid, phenylpropanoid, phenolic and other metabolism pathways were detected. Seven of 14 DEGs involved in terpene metabolism were down-regulated, while 19 of the 25 DEGs involved in flavonoid metabolism were up-regulated. Similar to DEGs involved in other metabolism pathways, most of the genes involved in phenylpropanoid and phenolic metabolism pathways were up-regulated. One gene involved in wax metabolism was induced. These data suggest that flagellin might enhance defense responses mediated by secondary metabolism in rice.

Other metabolisms such as amino acid synthesis and degradation, nucleotide metabolism and nitrogen metabolism, were also affected. All DEGs involved in amino acid degradation were up-regulated, while DEGs involved in amino acid synthesis showed varied expression patterns. Only one down-regulated DEG involved in nitrogen metabolism, a gene encoding glutamate synthase, was identified.

### Identification of DEGs involved in defense and signaling in rice

**Figure 5:**
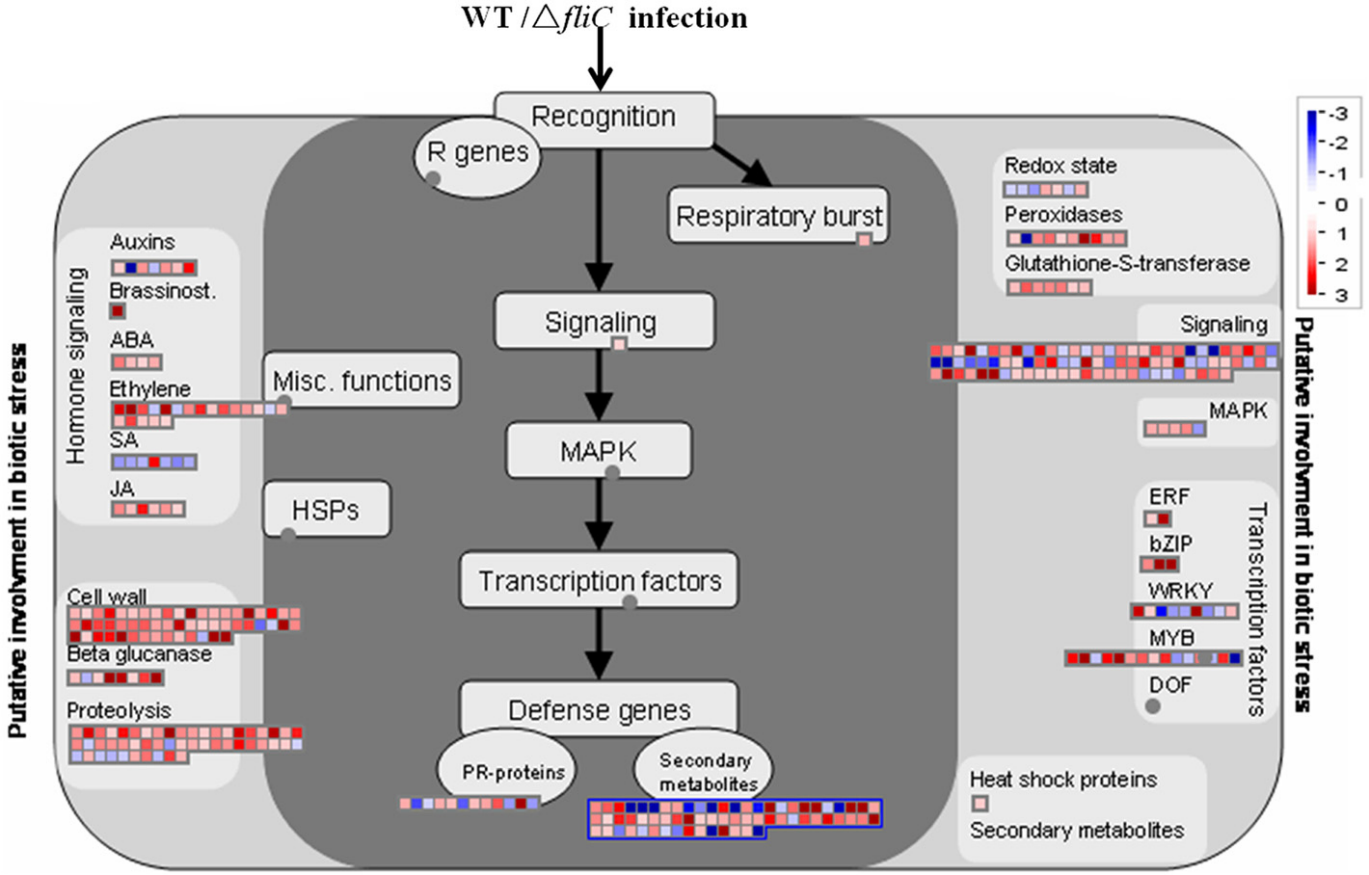
**MapMan visualization of defense response between WT and Δ** ***fliC*** **treatments.** 1,680 DGEs (fold changes ≥ 2, p-value ≤ 0.05) were imported into MapMan. DGEs up- and down-regulated are represented with red and blue squares, respectively. The grey circles means no differentially-expressed genes involved in this process. Values are log_2_-transformed fold changes.

To demonstrate the potential roles of bacterial flagellin in induction of defense responses in rice, the DEGs related to defense were particularly scrutinized between the WT and ∆*fliC* treatments. The DEGs included those with known functions in defense, such as TFs, PRs, kinases, peroxidases, etc. (Figure 5, Additional file 3: Table S3). DEGs encoding TFs, including ERF, bZIP, WRKY, MYB and DOF, were identified. Six of 12 DEGs encoding PRs were up-regulated, and the other six were down-regulated. Fifty one DEGs encoding kinase receptors were detected with expression levels influenced by WT and ∆*fliC* treatments. Nine of 10 DEGs encoding peroxidases and seven encoding glutathione-S-transferase were up-regulated.

DEGs involved in signaling of auxin, brassinosteriod, ABA, ethylene, salicylic acid, and jasmonic acid were revealed (Figure 5 and Additional file 4: Table S4). Compared with the ∆*fliC* treatment, five of seven DEGs associated with auxin synthesis, degradation and transport were up-regulated by the WT treatment, while two other DEGs were down-regulated. All DEGs involved in brassinosteriod, ABA and jasmonic acid synthesis and degradation were up-regulated. Six of seven DEGs associated with salicylic acid signaling pathways were down-regulated. In addition, 17 of 20 DEGs involved in ethylene signaling pathways were up-regulated.

### Identification of specifically-expressed genes in WT or ∆*fliC* treatments

**Figure 6:**
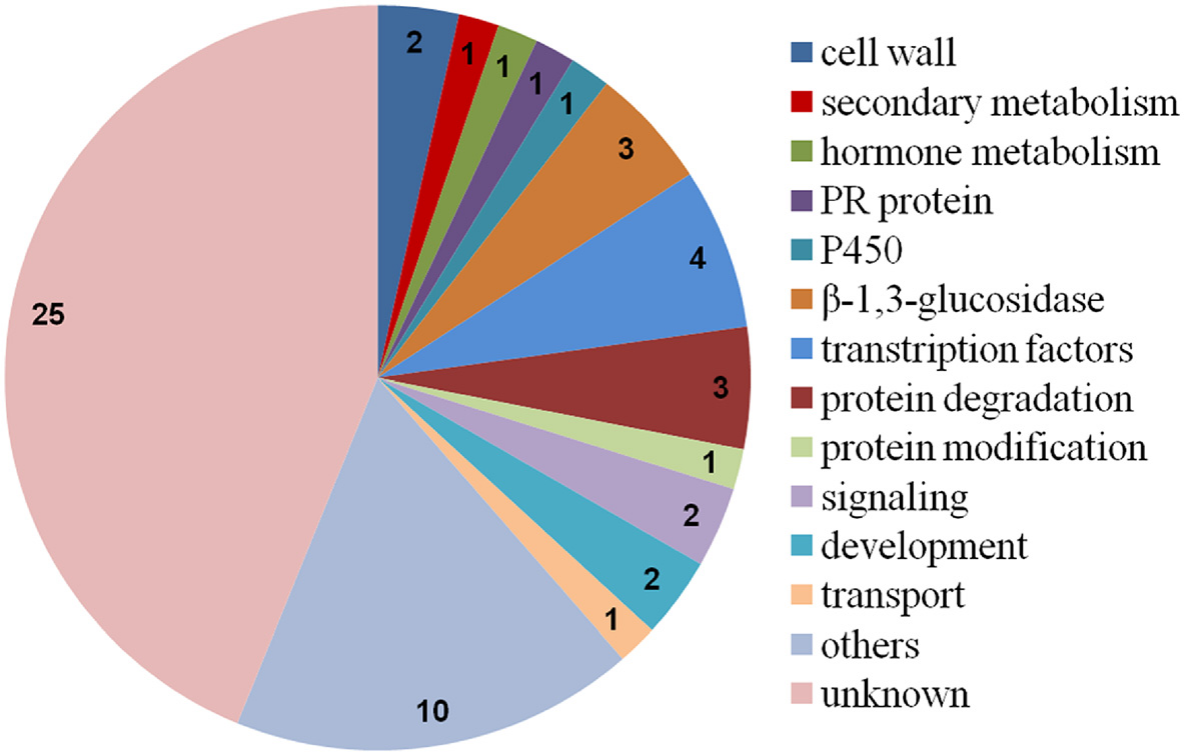
**Genes specifically expressed in the WT library.** Data represent the number of DEGs.

**Figure 7:**
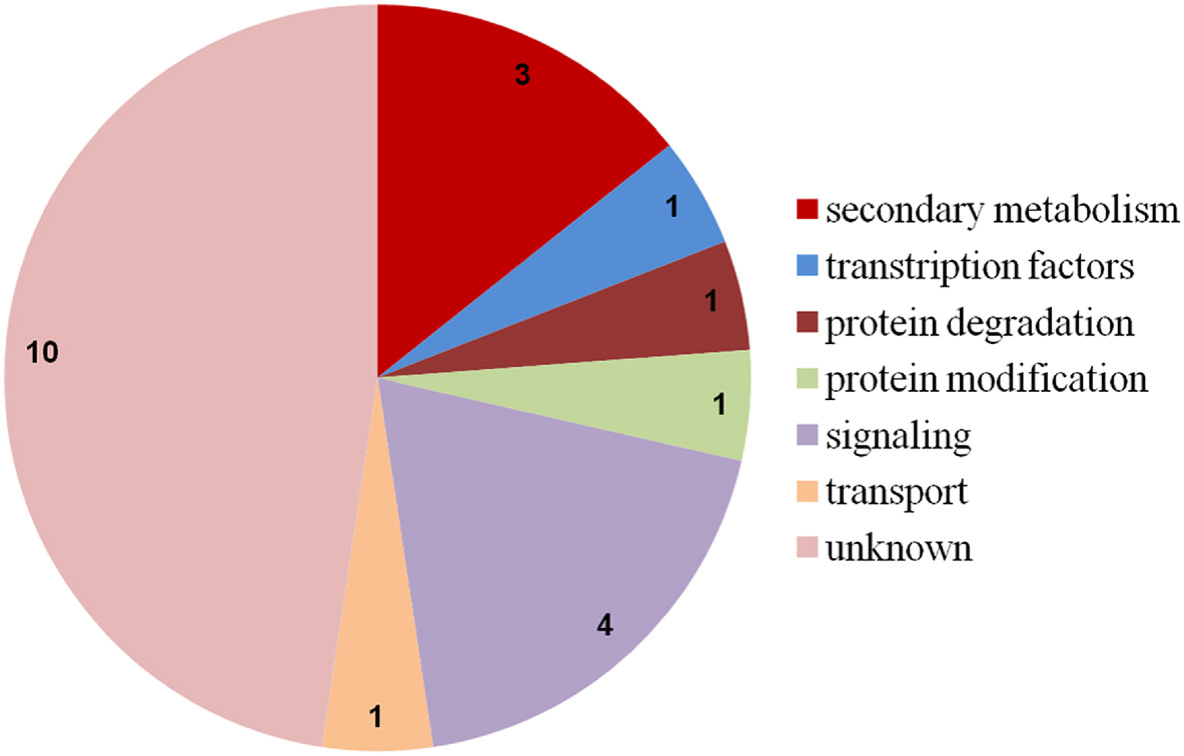
**Genes specifically expressed in the Δ** ***fliC*** **library.** Data represent the number of DEGs.

Fifty-seven specifically expressed genes (SEGs) were found after WT inoculation. The functions of SEGs were associated with the cell wall, secondary metabolism, hormone metabolism, protein degradation and modification, RNA transcription, signaling, development, transport, etc. (Figure 6 and Additional file 5: Table S6). In addition, 21 SEGs in the ∆*fliC*-treated library were identified to be involved in secondary metabolism, RNA transcription, protein degradation and modification, signaling and transport (Figure 7 and Additional file 6: Table S7).

## Discussion

Flagellin, the main component of flagella, not only plays an important role in bacterial motility, but it is also important in plant PTI (Lee et al. [2003]). Flagellin from avirulent but not virulent strains of the rice pathogen *Acidovorax avenae* induced hypersensitive cell death in cultured rice cells (Che et al. [2000]); the specificity of this induction of immune responses was attributed to flagellin glycosylation (Taguchi et al. [2010]; Hirai et al. [2011]), and suggested that the function of bacterial flagellin in PTI in rice relied on its origin and post-translational modification. We have shown that a *fliC* deletion mutant of Xoo causes increased disease (longer lesion lengths) in rice leaves relative to WT Xoo (Tian et al. [2014]). In this study, we identified DEGs by comparing transcriptomic responses in rice inoculated with the WT Xoo and *∆fliC*. The induction of *fliC* gene expression of Xoo during bacterial growth in rice indicated the expression levels of these DEGs were changed with flagellated Xoo invasion (Additional file 7: Figure S1). The putative functions of these DEGs revealed multiple biological processes, including cell wall synthesis, photosynthesis, defense response and hormone signaling, etc. are involved in rice responses to Xoo flagellin, and provide additional insights into the roles of bacterial flagellin played in the Xoo-rice interactions.

Expression of genes involved in cell wall formation and degradation were influenced by bacterial infection. Microbial pathogens enhance infection by degrading plant cell walls using cell-wall-degrading enzymes. On the other hand, cell wall degradation products produced by microbial activity can induce innate immune responses in plants, including callose deposition and programmed cell death (Jha et al. [2007]; Aparna et al. [2009]). In addition, some genes involved in cell wall modification play an important role in basal disease resistance (An et al. [2008]). Our current finding that up-regulation of DEGs associated with cell wall precursor synthesis, cell wall degradation and modification (Figure 4 and Additional file 2: Table S2) is consistent with other reports (Narsai et al. [2013]; Socquet-Juglard et al. [2013]).

PRs are synthesized during basal defense responses and enhanced disease resistance to pathogens in plants. Flagellin induces the expression of PR genes in *Arabidopsis* (Gomez-Gomez et al. [1999]). WRKY TFs form a large family of plant-specific transcription factors and can be positively or negatively regulated during defense responses (Chujo et al. [2013]). Constitutive expression of WRKY18 enhanced resistance to *Pseudomonas syringae* in *Arabidopsis*, while its co-expression with WRKY40 made plants more susceptible to *P. syringae* (Xu et al. [2006]; Schon et al. [2013]). Our results revealed seven up-regulated PRs involved in basal disease resistance, and down-regulated genes for WRKY18 and WRKY40 (Figure 5 and Additional file 3: Table S3), demonstrating these particular genes might play important roles in rice-Xoo interactions.

Pathogen infection usually results in the significant decreases of photosynthesis, and in turn, limits the availability of nutrient sources for pathogen (Berger et al. [2007]). For example, the photosynthesis was decreased in *Arabidopsis* leaves after challenged with *P. syringae* (Bonfig et al. [2006]). Genes encoding photosynthesis components were repressed by Xoo infection in rice (Narsai et al. [2013]). Activation of defense by PAMPs leads to rapid decrease in nonphotochemical quenching (Gohre et al. [2012]). Our data showed genes encoding proteins involved in light reactions, Calvin cycle and ATP synthesis were repressed in rice after treatment with WT Xoo relative to the mutant (Figure 4 and Additional file 4: Table S4), implying the bacterial flagellin might function in suppression of photosynthesis of rice.

Roles of salicylic acid (SA) and ethylene (ET)/jasmonic acid (JA)-mediated signaling pathways in resistance to pathogens have been well studied (Robert-Seilaniantz et al. [2011]). SA synthesis was accelerated by pathogen infection, and enhanced the induction of defense gene transcripts, H_2_O_2_ accumulation and hypersensitive cell death in plants (Gaffney et al. [1993]; Shirasu et al. [1997]), and ET/JA signaling pathway was required for resistance to necrotrophic pathogens(Glazebrook [2005]; Denance et al. [2013]). In addition, ET synthesis was induced by the PAMP flg22 (Felix et al. [1999]). In our study, several genes encoding 1-aminocyclopropane-1-carboxylate oxidase (ACO) and 12-oxophytodienoate reductase (OPR) were up-regulated (Figure 5 and Additional file 1: Table S5); these are key enzymes involved in ET and JA synthesis pathway, respectively (Vriezen et al. [1999]; Schaller et al. [2000]). These results indicated ET/JA synthesis were induced by Xoo flagellin in rice. Interestingly, seven genes encoding salicylic acid carboxyl methyltransferase (SAMT), catalyzing SA to methyl SA or dimethyl SA (Fukami et al. [2002]), were down-regulated. We suggest that SA accumulation in rice is induced by Xoo flagellin. Genes involved in auxin and brassinosteroid signaling pathways were also identified, but the relationship of these hormones and plant defense response is largely unknown.

## Conclusion

A number of rice genes differentially expressed after inoculation with WT Xoo relative to ∆*fliC* was identified by DGE analysis, some of which were validated by qRT-PCR assays for their expression patterns. These DEGs were involved in the multiple different biological functions, including induction of cell wall and lipid synthesis, secondary metabolism, defense responses, hormone signaling and suppression of photosynthesis. This study provided additional insights into molecular basis of rice response to bacterial infection and revealed potential functions of bacterial flagellin in the rice-Xoo interactions. Functional characterization is further required for these candidate genes of rice via the transgenic approaches including the gene over-expression and/or silencing analysis.

## Methods

### Plant materials and bacterial inoculation treatment

Rice seeds (*Oryza sativa* L. cv. Nipponbare) were germinated for 2 days in water at 37°C. Individual germinated seeds were planted in a small pots containing vermiculite, the pots were placed in a tray that contained 1 L Hogland’s solution, which was a mixture of 10 mM KH_2_PO_4_, 2 mM MgSO_4_, 1 mM CaCl_2_, 0.1 mM Fe-EDTA, 50 μM H_3_BO_4_, 12 μM MnSO_4_, 1 μM ZnCl_2_, 1 μM CuSO_4_, 0.2 μM Na_2_MoO_4_ and 3 mM KNO_3._ The pH of the nutrient solution was adjusted to 5.5-6 using 50% phosphoric acid, and the solution was replaced every two days. Growth conditions were 16 h light (29°C) and 8 h of dark (23°C). Xoo strain PXO99^A^ (Hopkins et al. [1992]) is a virulent strain to Nipponbare. ∆*fliC*, which is a *fliC* deletion mutant derived from PXO99^A^, was generated by marker exchange strategy as previous described (Tian et al. [2014]). PXO99^A^ and ∆*fliC* were grown for 72 h at 28°C in M210 media (Yang et al. [2012]), then the cells were collected by centrifugation and resuspended in d_2_H_2_O at an OD_600_ of 0.8 (approximately 1.0 × 10^8^ CFU/mL). Thirty day-old seedlings of rice were inoculated with PXO99^A^ and ∆*fliC* by the leaf-clipping method (Chen et al. [2002]). Five centimeters tip of inoculated rice leaves were collected at 12 h after inoculation, frozen in liquid nitrogen, and stored at −80°C.

In order to detect the *fliC* expression of during Xoo growth in rice, we collected the rice leaves, which were inoculated with PXO99^A^ at 7 d after inoculation. Then total RNA was extracted using Trizol reagent (Invitrogen, USA), and treated with DNase. The first strand cDNA fragment of *fliC* was synthesized using Superscript III reverse transcriptase (Invitrogen, USA). Specific primer sets, fliC-F (5′ –CCGAGCGTTTCACTACCCA– 3′) and fliC-R (5′ –ATCCTTGAACGACAGGCTGAT– 3′) were designed based on the sequence of *fliC*. PCR was performed with one denaturation cycle of 5 min at 98°C and 32 cycles of 30 s at 95°C, 30 s at 55°C, and 30 s at 72°C, then 10 min at 72°C. Ten microlitres of the PCR product was loaded onto 2% agarose gel. The PCR was performed without Superscript III reverse transcriptase as a negative control.

### Solexa/Illumina sequencing

Total RNA was extracted from rice leaves in WT treatment and ∆*fliC* treatment using Trizol reagent (Invitrogen). According to the manufacturer’s instruction of Solexa/Illumina sequencing, mRNA were isolated and enriched from 6 μg total RNA by using the oligo(dT) magnetic beads, then the first- and second-strand cDNA were synthesized by oligo(dT) primer. cDNA were digested with *Nla*III, and the cDNA fragments were ligated with adapter 1, digested with *Mme*I to produce tags with adaptor 1. After removing the magnetic beads, Illumina adaptor 2 was ligated to the 3′ ends of cDNA fragments, and a tag library was formed by different adaptors at both ends of fragments. Lastly, the library fragments were amplified by PCR and purified, and then sequenced via Illumina HiSeq™ 2000.

### Identification of differentially-expressed genes

Raw data from Solexa/Illumina sequencing were cleaned by removing reads containing the adapter or poly-N, orlow quality reads. At the same time, the Q20 content and GC content and sequence duplication level of the clean data were calculated. All downstream analyses were based on the clean data with high quality. All clean tags were mapped to reference sequences using bowtie software and allowing 2-bp mismatch. The number of clean tags for each gene was calculated and then normalized to reads per kilobase of exon model per million mapped reads (FPKM). Differentially expressed genes between two samples were identified by tophat and cufflinks softwares (Trapnell et al. [2009]; Roberts et al. [2011]), The P ≤ 0.05 and the absolute value of log_2_ Ratio ≥ 1 were chosen as the threshold to judge the significance of gene expression difference.

### Functional annotation of DEGs

Gene Ontology (GO) (Ashburner et al. [2000]) and MapMan (Thimm et al. [2004]) were used to analysis biological function of DEGs. The GO enrichment analysis was applied to describe product characteristics and reaction network of DEGs. All DEGs were mapped to GO terms in the database (http://bioinfo.cau.edu.cn/agriGO/). GO terms with corrected P value less than 0.05 were considered significantly enriched by differential expressed genes. MapMan software was used to visualize the expression change levels of individual genes in diagrams of metabolic pathways. As previously described (Thimm et al. [2004]), an *Oryza sativa* map was developed and uploaded to MapMan, and the change in expression ratio of each gene was calculated as the log_2_-fold change to generate the MapMan experimental file.

### Quantitative real-time PCR (qRT-PCR) analysis

Analysis of qRT-PCR were carried out using SYBR Green detection reagents (Quanta Biosciences, USA) in Applied Biosystem’s 7500 Sequence Detection System (Applied Biosystems, USA), and the 20 μl PCR reaction contained about 100 ng of cDNA. The reaction mixture was incubated at 95°C for 3 min, and then 40 cycles of 95°C for 10 s, 60°C for 30s, followed by a disassociation stage. Twelve DEGs were randomly selected, and specific primers of these genes were designed with the software Primer Premier 5.0 (PREMIER Biosoft Int., USA) (Additional file 8: Table S1). The rice actin gene was selected as a reference gene in qRT-PCR. The relative expression ratio was calculated using 2^-ΔΔCt^ method (Schmittgen and Livak [2008]). All samples were performed in three biological replicates and triplicate PCR, and the error bars show standard error.

## Competing interests

The authors declare that they have no competing interests.

## Author contributions

Conceived and designed the experiments: CY HMC CYH. Performed the experiments: CY HMC. Analyzed the data: CY HMC FT JEL CYH. Contributed reagents/materials/analysis tools: HMC FT. Wrote the paper: CY HMC FT JEL CYH. All authors read and approved the final manuscript.

## Additional files

## Supplementary Material

Additional file 1: Table S5. DEGs involved in photosynthesis. Click here for file

Additional file 2: Table S2. DEGs involved in cell wall. Click here for file

Additional file 3: Table S3. DEGs involved in defense response. Click here for file

Additional file 4: Table S4. DEGs involved in hormone signaling. Click here for file

Additional file 5: Table S6. DEGs specially expressed in WT library. Click here for file

Additional file 6: Table S7. DEGs specially expressed in ∆*fliC* library. Click here for file

Additional file 7: Figure S1. The expression level of *fliC* was detected during Xoo growth in-rice. WT: *fliC* expression of PXO99^A^ during growth in-rice; N: negative control. Click here for file

Additional file 8: Table S1. Primer sequences used for the validation of DEGs. Click here for file
