# Supplementary figures and images for: Differentially-expressed genes in rice infected by Xanthomonas oryzae pv. oryzae relative to a flagellin-deficient mutant reveal potential functions of flagellin in host–pathogen interactions
Source: Rice (N Y). 2014 Sep 3;7(1):20. doi: 10.1186/s12284-014-0020-7 (PMC4152760; doi:10.1186/s12284-014-0020-7)

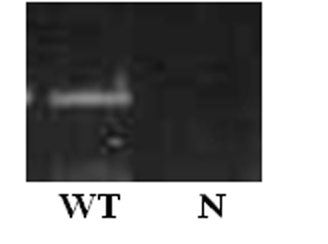

Supplement: Additional file 7: Figure S1. — The expression level of fliC was detected during Xoo growth in-rice. WT: fliC expression of PXO99A during growth in-rice; N: negative control. [file s12284-014-0020-7-S7.tiff]
